# Aqua­[2-(5-ethyl-2-pyridyl-κ*N*)-4-iso­propyl-4-methyl-5-oxo-4,5-dihydroxy­imidazol-1-ido-κ*N* ^1^](5-methyl-1*H*-pyrazole-3-carboxyl­ato-κ^2^ *N* ^2^,*O*)copper(II) 1.33-hydrate

**DOI:** 10.1107/S1600536809053768

**Published:** 2009-12-19

**Authors:** Ji-Chang Zhuang, Fei-Long Hu, Zhong-Jing Huang, Yue Zhuang, Feng Zhang

**Affiliations:** a College of Chemistry and Ecological Engineering, Guangxi University for Nationalities, Nanning 530006, People’s Republic of China

## Abstract

In the title complex, [Cu(C_5_H_5_N_2_O_2_)(C_14_H_18_N_3_O)(H_2_O)]·1.33H_2_O, the Cu^II^ ion is coordinated in a slightly distorted square-pyramidal environment. The basal plane is formed by two N atoms from a 2-(5-ethyl-2-pyridyl-κ*N*)-4-isopropyl-4-methyl-5-oxo-4,5-dihydroxy­imidazol-1-ide ligand and by one O atom and one N atom from a 5-methyl-1*H*-pyrazole-3-carboxyl­ate ligand. The apical position is occupied by a water mol­ecule. In the crystal structure, O—H⋯O, O—H⋯N and N—H⋯O hydrogen bonds lead to a three-dimensional supra­molecular network.

## Related literature

For general background to pyrazole and pyridine derivatives, see: Manna *et al.* (1992 ▶); Montoya *et al.* (2007 ▶); Perevalov *et al.* (2001 ▶).
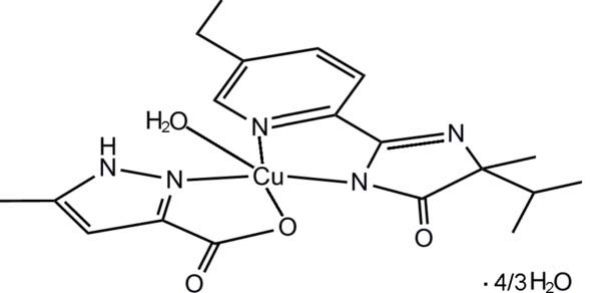


## Experimental

### 

#### Crystal data

[Cu(C_5_H_5_N_2_O_2_)(C_14_H_18_N_3_O)(H_2_O)]·1.33H_2_O
*M* *_r_* = 475.01
Trigonal, 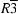

*a* = 26.7859 (3) Å
*c* = 16.6531 (5) Å
*V* = 10347.6 (4) Å^3^
*Z* = 18
Mo *K*α radiation
μ = 0.99 mm^−1^
*T* = 296 K
0.50 × 0.40 × 0.35 mm

#### Data collection

Bruker APEXII CCD diffractometer
Absorption correction: multi-scan (*SADABS*; Sheldrick, 1996 ▶) *T*_min_ = 0.638, *T*_max_ = 0.723
19606 measured reflections
5619 independent reflections
3406 reflections with *I* > 2σ(*I*)
*R*_int_ = 0.021

#### Refinement

*R*[*F* ^2^ > 2σ(*F* ^2^)] = 0.048
*wR*(*F* ^2^) = 0.168
*S* = 1.04
5619 reflections
279 parameters
H-atom parameters constrained
Δρ_max_ = 0.56 e Å^−3^
Δρ_min_ = −0.29 e Å^−3^

### 

Data collection: *APEX2* (Bruker, 2007 ▶); cell refinement: *SAINT* (Bruker, 2007 ▶); data reduction: *SAINT*; program(s) used to solve structure: *SHELXS97* (Sheldrick, 2008 ▶); program(s) used to refine structure: *SHELXL97* (Sheldrick, 2008 ▶); molecular graphics: *DIAMOND* (Brandenburg, 1999 ▶); software used to prepare material for publication: *SHELXTL* (Sheldrick, 2008 ▶).

## Supplementary Material

Crystal structure: contains datablocks I, global. DOI: 10.1107/S1600536809053768/hy2262sup1.cif

Structure factors: contains datablocks I. DOI: 10.1107/S1600536809053768/hy2262Isup2.hkl

Additional supplementary materials:  crystallographic information; 3D view; checkCIF report

## Figures and Tables

**Table 1:** Selected bond lengths (Å)

Cu1—N1	1.962 (2)
Cu1—N3	1.946 (3)
Cu1—N5	2.008 (2)
Cu1—O4	1.973 (2)
Cu1—O6	2.265 (2)

**Table 2:** Hydrogen-bond geometry (Å, °)

*D*—H⋯*A*	*D*—H	H⋯*A*	*D*⋯*A*	*D*—H⋯*A*
O1—H1⋯O2	0.85	2.12	2.965 (10)	172
O2—H2*A*⋯O5	0.85	2.08	2.838 (7)	148
O2—H2*B*⋯O2^i^	0.85	2.41	3.246 (10)	168
O6—H6*A*⋯O3^ii^	0.85	2.14	2.807 (3)	135
O6—H6*B*⋯N4^iii^	0.85	2.07	2.861 (3)	154
N2—H2⋯O5	0.83	2.01	2.733 (3)	144

Symmetry codes: (i) 
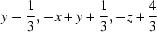
; (ii) 
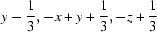
; (iii) 
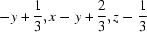
.

**Fig. 1.**
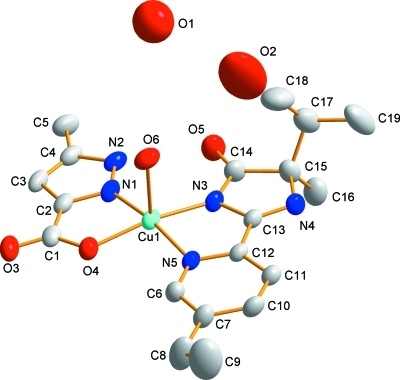
Molecular structure of the title compound, showing 30% probability displacement ellipsoids. H atoms have been omitted for clarity.

**Fig. 2.**
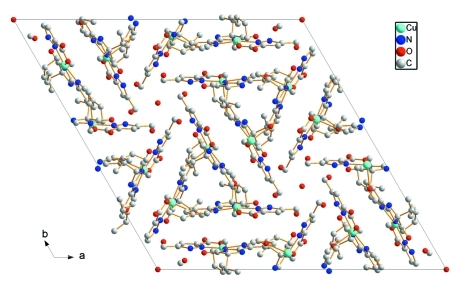
The crystal packing of the title compound, viewed down the c axis.

## supplementary crystallographic information

### Comment

The chemical and pharmacological properties of heterocyclic derivatives, particularly pyrazole and pyridine derivatives have been investigated extensively because of their chelating ability with metal ions and their potentially beneficial chemical and biological activities (Manna *et al.*, 1992; Montoya *et al.*, 2007; Perevalov *et al.*, 2001). During our research of these types of compounds, a new mixed-ligand copper(II) complex has been synthesized and characterized by single-crystal X-ray diffraction.

As illustrated in Fig. 1, the Cu^II^ ion is five-coordinated by three N atoms and two O atoms in a distorted square-pyramidal geometry (Table 1). The basal plane is formed by two N atoms from a 2-(5-ethylpyridin-2-yl)-5-isopropyl-5-methyl-imidazol-4-one ligand and by one O atom and one N atom from a 5-methyl-1*H*-pyrazole-3-carboxylate ligand. The apical position is occupied by the O atom from a water molecule. The complex molecules and uncoordinated water molecules are held together by hydrogen bonds (Table 2), generating a three-dimensional supramolecular network (Fig. 2).

### Experimental

All reagents were available commercially and were used without further purification. A mixture of 5-methyl-1*H*-pyrazole-3-carboxylatic acid (0.126 g, 1.0 mmol), 2-(5-ethyl-pyridin-2-yl)-5-isopropyl-5-methyl-3,5-dihydro-imidazol-4-one (0.245 g, 1.0 mmol), CuCl_2_.2H_2_O (0.170 g, 1.0 mmol), EtOH (10 ml) and H_2_O (10 ml) was sealed in a 25 ml Teflon-lined bomb and heated to 393 K for 3 d, and then cooled to room temperature. Blue crystals were obtained (yield 32% based on Cu).

### Refinement

H atoms were positioned geometrically and refined as riding atoms, with C—H = 0.93 (aromatic), 0.98 (CH), 0.97 (CH_2_) and 0.96 (CH_3_) Å and N—H= 0.83 Å, and with *U*_iso_(H) = 1.2*U*_eq_(C,*N*). H atoms of water molecules were located in a difference Fourier map and refined using a riding model, with O—H = 0.85 Å and with *U*_iso_(H) = 1.2*U*_eq_(O).

### Crystal data

**Table:**

[Cu(C_5_H_5_N_2_O_2_)(C_14_H_18_N_3_O)(H_2_O)]·1.33H_2_O	*D*_x_ = 1.372 Mg m^−^^3^
*M**_r_* = 475.01	Mo *K*α radiation, λ = 0.71073 Å
Trigonal, *R*3	Cell parameters from 6164 reflections
Hall symbol: -R 3	θ = 2.6–22.1°
*a* = 26.7859 (3) Å	µ = 0.99 mm^−^^1^
*c* = 16.6531 (5) Å	*T* = 296 K
*V* = 10347.6 (4) Å^3^	Block, blue
*Z* = 18	0.50 × 0.40 × 0.35 mm
*F*(000) = 4470	

### Data collection

**Table:**

Bruker APEXII CCD diffractometer	5619 independent reflections
Radiation source: fine-focus sealed tube	3406 reflections with *I* > 2σ(*I*)
graphite	*R*_int_ = 0.021
φ and ω scans	θ_max_ = 28.3°, θ_min_ = 1.5°
Absorption correction: multi-scan (*SADABS*; Sheldrick, 1996)	*h* = −33→27
*T*_min_ = 0.638, *T*_max_ = 0.723	*k* = −27→34
19606 measured reflections	*l* = −21→14

### Refinement

**Table:**

Refinement on *F*^2^	Primary atom site location: structure-invariant direct methods
Least-squares matrix: full	Secondary atom site location: difference Fourier map
*R*[*F*^2^ > 2σ(*F*^2^)] = 0.048	Hydrogen site location: inferred from neighbouring sites
*wR*(*F*^2^) = 0.168	H-atom parameters constrained
*S* = 1.04	*w* = 1/[σ^2^(*F*_o_^2^) + (0.0901*P*)^2^ + 5.4824*P*] where *P* = (*F*_o_^2^ + 2*F*_c_^2^)/3
5619 reflections	(Δ/σ)_max_ = 0.001
279 parameters	Δρ_max_ = 0.56 e Å^−^^3^
0 restraints	Δρ_min_ = −0.29 e Å^−^^3^

### Fractional atomic coordinates and isotropic or equivalent isotropic displacement parameters (Å^2^)

**Table:**

	*x*	*y*	*z*	*U*_iso_*/*U*_eq_	Occ. (<1)
Cu1	0.150787 (15)	0.398671 (15)	0.29814 (2)	0.05208 (17)	
N1	0.22614 (10)	0.46945 (11)	0.29242 (18)	0.0577 (7)	
N2	0.26293 (11)	0.51163 (11)	0.34201 (18)	0.0604 (7)	
H2	0.2578	0.5141	0.3908	0.072*	
N3	0.14042 (10)	0.39308 (10)	0.41407 (17)	0.0538 (7)	
N4	0.07626 (11)	0.33959 (11)	0.51314 (18)	0.0597 (7)	
N5	0.08268 (10)	0.31844 (10)	0.30232 (16)	0.0489 (6)	
O1	0.3333	0.6667	0.5264 (7)	0.246 (5)	
H1	0.3091	0.6352	0.5486	0.296*	0.67
O2	0.2584 (3)	0.5558 (3)	0.6093 (5)	0.326 (5)	
H2B	0.2449	0.5704	0.6420	0.391*	
H2A	0.2311	0.5303	0.5811	0.391*	
O3	0.22982 (10)	0.44486 (11)	0.08578 (16)	0.0709 (7)	
O4	0.16677 (9)	0.39531 (9)	0.18320 (14)	0.0607 (6)	
O5	0.20666 (10)	0.47281 (11)	0.48519 (16)	0.0834 (8)	
O6	0.09369 (10)	0.43745 (10)	0.27716 (15)	0.0698 (7)	
H6B	0.0659	0.4183	0.2450	0.084*	
H6A	0.1170	0.4717	0.2620	0.084*	
C1	0.21357 (13)	0.43775 (15)	0.1558 (2)	0.0572 (8)	
C2	0.24916 (13)	0.48057 (13)	0.2185 (2)	0.0556 (8)	
C3	0.30220 (13)	0.53166 (14)	0.2216 (3)	0.0628 (10)	
H3	0.3273	0.5496	0.1789	0.075*	
C4	0.31003 (13)	0.54998 (14)	0.2988 (3)	0.0629 (9)	
C5	0.35886 (15)	0.60083 (15)	0.3385 (3)	0.0810 (12)	
H5A	0.3457	0.6091	0.3877	0.121*	
H5B	0.3732	0.6336	0.3034	0.121*	
H5C	0.3891	0.5926	0.3499	0.121*	
C6	0.05778 (14)	0.28263 (14)	0.2408 (2)	0.0586 (8)	
H6	0.0749	0.2944	0.1905	0.070*	
C7	0.00924 (15)	0.23014 (15)	0.2462 (2)	0.0664 (9)	
C8	−0.0143 (2)	0.1906 (2)	0.1730 (3)	0.1164 (19)	
H8A	−0.0025	0.2145	0.1252	0.140*	
H8B	0.0037	0.1670	0.1710	0.140*	
C9	−0.0737 (3)	0.1542 (3)	0.1694 (4)	0.172 (3)	
H9A	−0.0856	0.1260	0.2114	0.257*	
H9B	−0.0840	0.1351	0.1182	0.257*	
H9C	−0.0926	0.1764	0.1761	0.257*	
C10	−0.01637 (15)	0.21426 (13)	0.3225 (2)	0.0626 (9)	
H10	−0.0504	0.1793	0.3291	0.075*	
C11	0.00827 (13)	0.24971 (13)	0.3871 (2)	0.0539 (8)	
H11	−0.0081	0.2390	0.4379	0.065*	
C12	0.05862 (12)	0.30228 (12)	0.37493 (19)	0.0464 (7)	
C13	0.08981 (12)	0.34381 (12)	0.4392 (2)	0.0486 (7)	
C14	0.16219 (14)	0.42548 (15)	0.4813 (2)	0.0642 (9)	
C15	0.12202 (15)	0.39227 (15)	0.5514 (2)	0.0689 (10)	
C16	0.15575 (18)	0.37754 (19)	0.6134 (3)	0.0885 (12)	
H16A	0.1319	0.3596	0.6595	0.133*	
H16B	0.1898	0.4123	0.6296	0.133*	
H16C	0.1665	0.3516	0.5896	0.133*	
C17	0.09624 (19)	0.42720 (19)	0.5869 (3)	0.0949 (14)	
H17	0.1278	0.4613	0.6127	0.114*	
C18	0.0717 (3)	0.4477 (2)	0.5255 (4)	0.132 (2)	
H18A	0.0469	0.4163	0.4910	0.198*	
H18B	0.1023	0.4775	0.4944	0.198*	
H18C	0.0499	0.4629	0.5509	0.198*	
C19	0.0519 (2)	0.3941 (2)	0.6506 (3)	0.132 (2)	
H19A	0.0405	0.4190	0.6760	0.198*	
H19B	0.0682	0.3802	0.6901	0.198*	
H19C	0.0189	0.3621	0.6265	0.198*	

### Atomic displacement parameters (Å^2^)

**Table:**

	*U*^11^	*U*^22^	*U*^33^	*U*^12^	*U*^13^	*U*^23^
Cu1	0.0392 (2)	0.0468 (2)	0.0638 (3)	0.01661 (16)	0.00573 (17)	0.01295 (18)
N1	0.0401 (13)	0.0552 (16)	0.0731 (19)	0.0204 (12)	0.0081 (13)	0.0200 (14)
N2	0.0430 (14)	0.0435 (14)	0.090 (2)	0.0179 (12)	−0.0019 (14)	0.0079 (14)
N3	0.0410 (13)	0.0460 (14)	0.0623 (18)	0.0127 (11)	0.0032 (12)	0.0099 (13)
N4	0.0491 (15)	0.0518 (15)	0.0628 (19)	0.0137 (12)	0.0089 (13)	0.0010 (13)
N5	0.0450 (13)	0.0447 (13)	0.0545 (16)	0.0206 (11)	0.0016 (12)	0.0094 (12)
O1	0.255 (8)	0.255 (8)	0.229 (12)	0.127 (4)	0.000	0.000
O2	0.278 (9)	0.199 (7)	0.314 (9)	−0.021 (6)	0.021 (7)	−0.127 (7)
O3	0.0568 (14)	0.0923 (18)	0.0720 (17)	0.0436 (13)	0.0174 (12)	0.0276 (14)
O4	0.0458 (12)	0.0623 (14)	0.0677 (15)	0.0223 (11)	0.0077 (11)	0.0128 (11)
O5	0.0602 (15)	0.0625 (15)	0.0893 (19)	0.0020 (12)	0.0027 (14)	−0.0104 (14)
O6	0.0606 (13)	0.0581 (13)	0.0934 (18)	0.0317 (11)	−0.0160 (13)	0.0076 (12)
C1	0.0462 (17)	0.068 (2)	0.069 (2)	0.0373 (16)	0.0083 (17)	0.0217 (18)
C2	0.0415 (15)	0.0525 (18)	0.078 (2)	0.0272 (14)	0.0076 (16)	0.0224 (17)
C3	0.0404 (16)	0.0566 (19)	0.094 (3)	0.0260 (15)	0.0203 (18)	0.0316 (19)
C4	0.0404 (16)	0.0477 (18)	0.101 (3)	0.0225 (14)	0.0122 (18)	0.0194 (19)
C5	0.052 (2)	0.051 (2)	0.128 (4)	0.0171 (16)	0.007 (2)	0.005 (2)
C6	0.063 (2)	0.0554 (19)	0.054 (2)	0.0271 (16)	0.0028 (16)	0.0069 (16)
C7	0.062 (2)	0.055 (2)	0.067 (2)	0.0179 (17)	−0.0054 (18)	0.0076 (17)
C8	0.111 (4)	0.083 (3)	0.085 (3)	−0.005 (3)	−0.019 (3)	−0.009 (3)
C9	0.135 (6)	0.166 (6)	0.135 (6)	0.015 (5)	−0.027 (4)	−0.027 (5)
C10	0.0569 (19)	0.0419 (17)	0.075 (2)	0.0145 (15)	−0.0043 (18)	0.0063 (17)
C11	0.0471 (16)	0.0472 (17)	0.062 (2)	0.0195 (14)	−0.0003 (15)	0.0085 (15)
C12	0.0396 (14)	0.0406 (15)	0.059 (2)	0.0200 (12)	0.0013 (14)	0.0106 (14)
C13	0.0389 (15)	0.0422 (15)	0.061 (2)	0.0173 (12)	0.0030 (14)	0.0091 (15)
C14	0.0512 (19)	0.0522 (19)	0.074 (2)	0.0142 (15)	0.0029 (17)	−0.0041 (18)
C15	0.056 (2)	0.061 (2)	0.069 (2)	0.0129 (16)	0.0051 (18)	−0.0118 (18)
C16	0.082 (3)	0.087 (3)	0.069 (3)	0.022 (2)	−0.010 (2)	−0.007 (2)
C17	0.071 (3)	0.077 (3)	0.113 (4)	0.020 (2)	0.015 (3)	−0.020 (3)
C18	0.137 (5)	0.106 (4)	0.180 (6)	0.080 (4)	0.022 (4)	−0.005 (4)
C19	0.081 (3)	0.125 (4)	0.146 (5)	0.018 (3)	0.036 (3)	−0.052 (4)

### Geometric parameters (Å, °)

**Table:**

Cu1—N1	1.962 (2)	C6—C7	1.359 (5)
Cu1—N3	1.946 (3)	C6—H6	0.9300
Cu1—N5	2.008 (2)	C7—C10	1.405 (5)
Cu1—O4	1.973 (2)	C7—C8	1.529 (6)
Cu1—O6	2.265 (2)	C8—C9	1.391 (7)
N1—C2	1.341 (4)	C8—H8A	0.9700
N1—N2	1.348 (4)	C8—H8B	0.9700
N2—C4	1.367 (4)	C9—H9A	0.9600
N2—H2	0.8300	C9—H9B	0.9600
N3—C14	1.357 (4)	C9—H9C	0.9600
N3—C13	1.402 (3)	C10—C11	1.367 (5)
N4—C13	1.272 (4)	C10—H10	0.9300
N4—C15	1.473 (4)	C11—C12	1.394 (4)
N5—C6	1.332 (4)	C11—H11	0.9300
N5—C12	1.336 (4)	C12—C13	1.467 (4)
O1—H1	0.8500	C14—C15	1.535 (5)
O2—H2B	0.8501	C15—C17	1.532 (6)
O2—H2A	0.8501	C15—C16	1.547 (6)
O3—C1	1.226 (4)	C16—H16A	0.9600
O4—C1	1.283 (4)	C16—H16B	0.9600
O5—C14	1.233 (4)	C16—H16C	0.9600
O6—H6B	0.8498	C17—C18	1.462 (7)
O6—H6A	0.8499	C17—C19	1.506 (6)
C1—C2	1.490 (5)	C17—H17	0.9800
C2—C3	1.396 (4)	C18—H18A	0.9600
C3—C4	1.355 (5)	C18—H18B	0.9600
C3—H3	0.9300	C18—H18C	0.9600
C4—C5	1.490 (5)	C19—H19A	0.9600
C5—H5A	0.9600	C19—H19B	0.9600
C5—H5B	0.9600	C19—H19C	0.9600
C5—H5C	0.9600		
			
N3—Cu1—N1	99.25 (11)	C9—C8—H8B	108.1
N3—Cu1—O4	170.05 (10)	C7—C8—H8B	108.1
N1—Cu1—O4	81.68 (11)	H8A—C8—H8B	107.3
N3—Cu1—N5	82.23 (10)	C8—C9—H9A	109.5
N1—Cu1—N5	168.82 (10)	C8—C9—H9B	109.5
O4—Cu1—N5	94.97 (10)	H9A—C9—H9B	109.5
N3—Cu1—O6	94.89 (10)	C8—C9—H9C	109.5
N1—Cu1—O6	98.84 (10)	H9A—C9—H9C	109.5
O4—Cu1—O6	94.75 (9)	H9B—C9—H9C	109.5
N5—Cu1—O6	92.05 (9)	C11—C10—C7	120.6 (3)
C2—N1—N2	108.3 (3)	C11—C10—H10	119.7
C2—N1—Cu1	113.3 (2)	C7—C10—H10	119.7
N2—N1—Cu1	138.4 (2)	C10—C11—C12	118.3 (3)
N1—N2—C4	108.7 (3)	C10—C11—H11	120.9
N1—N2—H2	125.8	C12—C11—H11	120.9
C14—N3—C13	105.1 (3)	N5—C12—C11	121.7 (3)
C14—N3—Cu1	140.4 (2)	N5—C12—C13	114.5 (2)
C13—N3—Cu1	113.7 (2)	C11—C12—C13	123.8 (3)
C13—N4—C15	105.7 (3)	N4—C13—N3	118.1 (3)
C6—N5—C12	118.4 (3)	N4—C13—C12	127.6 (3)
C6—N5—Cu1	127.1 (2)	N3—C13—C12	114.3 (3)
C12—N5—Cu1	114.3 (2)	O5—C14—N3	125.9 (3)
H2B—O2—H2A	109.3	O5—C14—C15	126.4 (3)
C1—O4—Cu1	116.2 (2)	N3—C14—C15	107.7 (3)
Cu1—O6—H6B	114.0	N4—C15—C17	109.8 (3)
Cu1—O6—H6A	104.0	N4—C15—C14	103.4 (3)
H6B—O6—H6A	114.4	C17—C15—C14	109.8 (3)
O3—C1—O4	126.1 (3)	N4—C15—C16	110.9 (3)
O3—C1—C2	120.4 (3)	C17—C15—C16	113.4 (4)
O4—C1—C2	113.5 (3)	C14—C15—C16	109.0 (3)
N1—C2—C3	108.3 (3)	C15—C16—H16A	109.5
N1—C2—C1	115.2 (3)	C15—C16—H16B	109.5
C3—C2—C1	136.5 (3)	H16A—C16—H16B	109.5
C4—C3—C2	106.6 (3)	C15—C16—H16C	109.5
C4—C3—H3	126.7	H16A—C16—H16C	109.5
C4—N2—H2	125.5	H16B—C16—H16C	109.5
C2—C3—H3	126.7	C18—C17—C19	110.1 (5)
C3—C4—N2	108.1 (3)	C18—C17—C15	112.6 (4)
C3—C4—C5	131.2 (3)	C19—C17—C15	112.1 (4)
N2—C4—C5	120.8 (4)	C18—C17—H17	107.2
C4—C5—H5A	109.5	C19—C17—H17	107.2
C4—C5—H5B	109.5	C15—C17—H17	107.2
H5A—C5—H5B	109.5	C17—C18—H18A	109.5
C4—C5—H5C	109.5	C17—C18—H18B	109.5
H5A—C5—H5C	109.5	H18A—C18—H18B	109.5
H5B—C5—H5C	109.5	C17—C18—H18C	109.5
N5—C6—C7	124.7 (3)	H18A—C18—H18C	109.5
N5—C6—H6	117.6	H18B—C18—H18C	109.5
C7—C6—H6	117.6	C17—C19—H19A	109.5
C6—C7—C10	116.3 (3)	C17—C19—H19B	109.5
C6—C7—C8	121.0 (3)	H19A—C19—H19B	109.5
C10—C7—C8	122.8 (3)	C17—C19—H19C	109.5
C9—C8—C7	116.8 (5)	H19A—C19—H19C	109.5
C9—C8—H8A	108.1	H19B—C19—H19C	109.5
C7—C8—H8A	108.1		
			
N3—Cu1—N1—C2	171.2 (2)	Cu1—N5—C6—C7	−175.2 (3)
O4—Cu1—N1—C2	1.2 (2)	N5—C6—C7—C10	1.6 (5)
N5—Cu1—N1—C2	74.4 (6)	N5—C6—C7—C8	−176.6 (4)
O6—Cu1—N1—C2	−92.3 (2)	C6—C7—C8—C9	−149.2 (6)
N3—Cu1—N1—N2	−8.8 (3)	C10—C7—C8—C9	32.7 (8)
O4—Cu1—N1—N2	−178.8 (3)	C6—C7—C10—C11	−2.1 (5)
N5—Cu1—N1—N2	−105.6 (6)	C8—C7—C10—C11	176.1 (4)
O6—Cu1—N1—N2	87.7 (3)	C7—C10—C11—C12	1.2 (5)
C2—N1—N2—C4	−0.1 (3)	C6—N5—C12—C11	−0.8 (4)
Cu1—N1—N2—C4	179.9 (2)	Cu1—N5—C12—C11	174.9 (2)
N1—Cu1—N3—C14	15.1 (4)	C6—N5—C12—C13	178.6 (3)
N5—Cu1—N3—C14	−176.1 (4)	Cu1—N5—C12—C13	−5.8 (3)
O6—Cu1—N3—C14	−84.7 (4)	C10—C11—C12—N5	0.3 (4)
N1—Cu1—N3—C13	−177.3 (2)	C10—C11—C12—C13	−179.1 (3)
N5—Cu1—N3—C13	−8.5 (2)	C15—N4—C13—N3	0.0 (4)
O6—Cu1—N3—C13	82.9 (2)	C15—N4—C13—C12	179.4 (3)
N3—Cu1—N5—C6	−176.8 (3)	C14—N3—C13—N4	−0.8 (4)
N1—Cu1—N5—C6	−78.3 (6)	Cu1—N3—C13—N4	−172.6 (2)
O4—Cu1—N5—C6	−6.4 (3)	C14—N3—C13—C12	179.7 (3)
O6—Cu1—N5—C6	88.6 (3)	Cu1—N3—C13—C12	7.9 (3)
N3—Cu1—N5—C12	8.0 (2)	N5—C12—C13—N4	179.3 (3)
N1—Cu1—N5—C12	106.4 (6)	C11—C12—C13—N4	−1.4 (5)
O4—Cu1—N5—C12	178.4 (2)	N5—C12—C13—N3	−1.3 (4)
O6—Cu1—N5—C12	−86.6 (2)	C11—C12—C13—N3	178.1 (3)
N1—Cu1—O4—C1	−2.7 (2)	C13—N3—C14—O5	−179.6 (4)
N5—Cu1—O4—C1	−171.9 (2)	Cu1—N3—C14—O5	−11.4 (6)
O6—Cu1—O4—C1	95.6 (2)	C13—N3—C14—C15	1.1 (4)
Cu1—O4—C1—O3	−176.6 (2)	Cu1—N3—C14—C15	169.3 (3)
Cu1—O4—C1—C2	3.4 (3)	C13—N4—C15—C17	117.8 (4)
N2—N1—C2—C3	−0.2 (3)	C13—N4—C15—C14	0.7 (4)
Cu1—N1—C2—C3	179.76 (19)	C13—N4—C15—C16	−116.0 (3)
N2—N1—C2—C1	−179.9 (2)	O5—C14—C15—N4	179.6 (4)
Cu1—N1—C2—C1	0.1 (3)	N3—C14—C15—N4	−1.2 (4)
O3—C1—C2—N1	177.7 (3)	O5—C14—C15—C17	62.4 (5)
O4—C1—C2—N1	−2.3 (4)	N3—C14—C15—C17	−118.3 (4)
O3—C1—C2—C3	−1.8 (5)	O5—C14—C15—C16	−62.4 (5)
O4—C1—C2—C3	178.2 (3)	N3—C14—C15—C16	116.9 (3)
N1—C2—C3—C4	0.5 (3)	N4—C15—C17—C18	−63.5 (5)
C1—C2—C3—C4	−180.0 (3)	C14—C15—C17—C18	49.6 (5)
C2—C3—C4—N2	−0.6 (3)	C16—C15—C17—C18	171.8 (4)
C2—C3—C4—C5	178.4 (3)	N4—C15—C17—C19	61.3 (5)
N1—N2—C4—C3	0.5 (3)	C14—C15—C17—C19	174.4 (4)
N1—N2—C4—C5	−178.6 (3)	C16—C15—C17—C19	−63.4 (5)
C12—N5—C6—C7	−0.2 (5)		

### Hydrogen-bond geometry (Å, °)

**Table:**

*D*—H···*A*	*D*—H	H···*A*	*D*···*A*	*D*—H···*A*
O1—H1···O2	0.85	2.12	2.965 (10)	172
O2—H2A···O5	0.85	2.08	2.838 (7)	148
O2—H2B···O2^i^	0.85	2.41	3.246 (10)	168
O6—H6A···O3^ii^	0.85	2.14	2.807 (3)	135
O6—H6B···N4^iii^	0.85	2.07	2.861 (3)	154
N2—H2···O5	0.83	2.01	2.733 (3)	144

Symmetry codes: (i) *y*−1/3, −*x*+*y*+1/3, −*z*+4/3; (ii) *y*−1/3, −*x*+*y*+1/3, −*z*+1/3; (iii) −*y*+1/3, *x*−*y*+2/3, *z*−1/3.
